# Comparative Study on Relationship Between Inconsistent Online-Offline Social Performance and Self-Efficacy of University Students Based on Types of Social Activity

**DOI:** 10.3389/fpsyg.2021.603971

**Published:** 2021-03-15

**Authors:** Yang Yang, Yan Dongdong, Hu Yu

**Affiliations:** ^1^Research Institute of Social Development, Southwestern University of Finance and Economics, Chengdu, China; ^2^Department of Sociology, School of Ethnology and Sociology, Inner Mongolia University, Hohhot, China

**Keywords:** university students, types of social activity, self-efficacy, inconsistent performance, online-offline social performance

## Abstract

Social behavior is closely linked to self-efficacy, which is the individual’s confidence or belief that they can successfully complete a task in a given situation. The advent of social media classified social behavior as online and offline sociality, and has cultivated inconsistency in online and offline social behavior of university students, an issue that has come to prominence in scholarly research. However, the relationship between this inconsistency and self-efficacy is worthy of investigation because this particular confluence of behavioral concepts has been rarely been researched. In this paper, online and offline social behavior is integrated, a typology for university student social activities established, and the correlation between different types of social activity and student self-efficacy investigated, with a specific focus on those with notable inconsistencies in their social performance. The following findings are reported. First, as online social networking has become the dominant form of social interaction, the types of social activity have increased, with one-third of university students showing inconsistent online and offline social behavior. However, different types of social activities have varied effects on the self-efficacy of university students, with differences between general self-efficacy, which is significantly above academic self-efficacy, and social self-efficacy. These effects are also different for students with inconsistent online and offline social performance; those who are active online show higher self-efficacy than those who are active offline. This study shows online social network interactions to be more closely related to student self-efficacy than offline interactions.

## Introduction

With the widespread use of social media, online networking has emerged as the important form of social interaction. Research on real-life social behavior has concluded that social activity is positively related to the self-efficacy of university students (Howie et al., 2015). Research on the relationship between self-efficacy and networking activities has received increasing attention (Zhang, 2015; Gong et al., 2017). Analyses of online social behavior have reached similar conclusions. However, online social behavior is not necessarily the continuance of offline social behavior. Students with an active offline social performance may not be active in the virtual space, and vice versa. Thus, this study was made to determine if the inconsistency between online and offline social activity is commonplace among university students. Specifically, it is aimed at determining if the self-efficacy of students with conflicting online and offline social behavior is distinct from those with consistent social behavior and, if so, to identify the differences and how to measure them. To address the propositions above, in this paper, the online and offline social behavior of university students is integrated, the types of social activity classified using two-dimensional indicators, and self-efficacy of the groups compared, with a special focus on those with inconsistent social behavior.

### Online and Offline Social Interactions Increase Types of Social Activity and Give Rise to Inconsistent Social Behavior

Online and offline social platforms have become a joint avenue for social interaction, shaping the self-efficacy of university students and giving rise to inconsistent social behavior. Before the advent of social media, social interactions taking place in physical space were an important source of self-efficacy (Bi and Huang, 2009; Fan et al., 2010), while social media has extended social life into the virtual space. Online social networking has become an integral part of students’ social lives and tends to have a reciprocal effect on self-efficacy. In several studies, it has been suggested that online behavior somewhat tends to mimic the behavior expected of one’s offline identity and personality (Patra et al., 2013); that is, online and offline social performance bear some relevance to each another, and users exhibiting social behavior with high levels of extroversion offline tend to have high self-efficacy and are more socially active online (Liu and Larose, 2008; Schredl and Gritz, 2019).

In the “rich-get-richer hypothesis” (Kraut et al., 2002), it is proposed that individuals with higher extroversion or who are more comfortable in social situations are more likely to extend their active and positive social performance to the virtual space. According to this hypothesis, for individuals who are extroverted and already have strong social skills, using the Internet predicts active online social interactions and more social support, which further augments their social performance. However, several studies indicate that a proportion of socia**l** media users exhibit inconsistent online and offline social behavior (Kyle, 2006; de Ziga et al., 2017). The “social compensation hypothesis” describes active users of social media as attempting to compensate for inadequate offline representation. The theory claims that the anonymous online environment enables those who possess inadequate social skills to engage more actively on social forums, making up for any perceived disadvantages in offline environments (Valkenburg et al., 2005). Follow-up empirical research backs these two theories separately.

**FIGURE 1:**
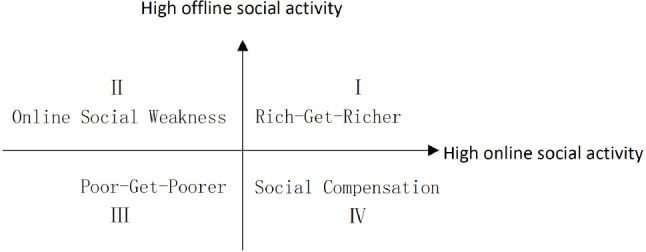
Four types of social activity.

However, the social compensation and rich-get-richer hypotheses cannot explain all of the differences in social activity. To develop a more robust typology to describe the dimensions of online and offline social performance, the horizontal axis is used herein to represent the activity of online social activity and the vertical axis to represent offline social activity. The resulting four quadrants represent four distinct types of social activity, including “Rich-Get-Richer” and “Social Compensation” (Figure 1). University students in the first quadrant are active in both online and offline social spheres; they fall into the “Rich-Get-Richer” category. Those in the fourth quadrant are active in online social activities but passive offline, and fit the parameters of the social compensation hypothesis.

Few studies touch on the other two groups in the second and third quadrants. For those who are active offline but passive online, we label the group of students with “online social weakness” relative to their active performance in offline social settings. In contrast to “Rich-Get-Richer,” those in the third quadrant exhibit passive social performance, both online and offline. Considering that their low online and offline social activity compounds their social disadvantages, the group is named herein “Poor-Get-Poorer.” These are the four types of social activity adopted in this paper to categorize university students, in which the online social weakness and social compensation groups show conflicting online and offline social behavior, and the “Rich-Get-Richer” and “Poor-Get-Poorer” groups demonstrate consistent online and offline behavior.

### Inconsistent Social Behavior and Self-Efficacy

Previous studies have confirmed the positive correlation between (online and offline) social activity and self-efficacy (Kramer and Winter, 2008; Hu et al., 2018). This is because the group that is not active in online social interaction often shows some negative emotions, which may hide the underlying information that individuals do not spend time to think and work hard, and their sense of self-efficacy is low (White et al., 2018). For those with consistent online and offline social behavior, their self-efficacy is positively related to their social activity: the “rich-get-richer” group achieves the highest self-efficacy and the “poor-get-poorer” group the lowest. For the other two groups, “social compensation” and “online social weakness,” their self-efficacy has not yet been rigorously examined. For university students who fall into these two socializing categories, the aim herein is to discover if their self-efficacy is more intertwined with online social interactions or, rather, with their offline behavior.

Self-efficacy is measured by a different aspect. “General self-efficacy” is the overall belief in one’s competence to approach novel tasks and to cope with adversity in a broad range of stressful or challenging encounters. The academic community has not yet reached a consensus on the corresponding relationship between general and specific self-efficacy although it is acknowledged that general self-efficacy is related to specific self-efficacy, and specific self-efficacy predicts more specific behavior. In this paper, the self-efficacy of university students is evaluated from the perspectives of general and specific self-efficacy. As studying and engaging in interpersonal relationships are some of the principal tasks of university students, their specific self-efficacy is mainly analyzed through academic and social self-efficacy.

## Materials and Methods

### Research Hypotheses

It is assumed in this work that online social interactions are more closely related to self-efficacy than similar offline interactions. This basic assumption is based on the idea that different types of social activity have a significant impact on university students’ self-efficacy. In descending order, and in terms of self-efficacy, “rich-get-richer” is assumed to be greater than “social compensation,” which is greater than “online social network advantaged,” which is greater than “poor-get-poorer.”

This set of basic assumptions translates into the following research hypotheses.

Hypothesis 1: In terms of general self-efficacy, the following is assumed: “rich get richer” > “social compensation” > “online social weakness” > “poor get poorer.”

Hypothesis 2: In terms of specific self-efficacy (academic and social self-efficacy), the categories are assumed to fall into the same order as general self-efficacy.

### Questionnaires

Data were acquired through a random sampling of questionnaires completed by students of Southwestern University of Finance and Economics, which entered the national 211 Project in southwestern China’s Sichuan Province. The questionnaire consisted of three parts. The first requested participants’ basic information, including sex, age, time spent online each day, self-perceived personality traits, household registration (eastern, central or western regions of China, and urban or rural areas), and their family circumstances (paternal education and household monthly income). The second and third parts were the focus of the questionnaire. The second part presented the scales of their online and offline social activity, and the third part covered the scales of self-efficacy.

Social activity is the social behavior pattern which can be seen as positive or negative according to one’s activeness. Social behavior contains relationship building and retention (Ran et al., 2017). As for young people, relationship building mainly includes image creation and dissemination. While the former refers to impression management that is strongly related to self-presentation, the latter involves sending out messages via online platforms and online or offline interactions with others. Relationship retention among college students includes sharing of feelings and opinions as well as social gatherings.

We use two 10-item seven-point Likert scales to measure offline and online social activity, respectively. The structure of two scales are similar – both including the relationship building and relationship retention. The total possible score of the scales reach 70 points, with higher scores indicative of a higher level of social activity.

For offline social activity, five items designed to assess the offline relationship building (e.g., “I care about my image,” “I play important role in many social activities”), and five items designed to assess the offline relationship retention (e.g., “I often share my life experiences with my friends” “My friends often share their feelings with me”).

For online social behavior, relationship building and retention can be indicated broadcasting and communication, respectively (Kim and Shen, 2020). Broadcasting was measured by six items (e.g., “The photos I post online are carefully selected”, “I often update my status on social media”). And online communication was measured by four items (e.g., “I often comment on or thumb up my friends posts,” “I like to communicate with others on social media”).

The scales of self-efficacy in the third part include the scales of general, academic, and social self-efficacy. Questions that are widely accepted under General Self-Efficacy Scale (GSES) guidelines were adopted, with each question measured on a five-point Likert scale with a total score of 50. Based on the GSES, the scale for task-specific efficacy was built with reference to academic self-efficacy as formulated by Wood and Locke (ASEQ) and social self-efficacy as developed by Xie and Zhang (2009). Each scale comprises eight questions, each measured on a seven-point Likert scale with a total score of 56. Regarding the other scales, the score is indicative of the level of specific task self-efficacy.

The reliability indexes for the offline social activity, online social activity, social self-efficacy, and academic self-efficacy scales of Cronbach’s alpha were 0.783, 0.789, 0.824, and 0.875, respectively, indicating a high degree of reliability.

### The Sampling and Data Analysis

The survey conducted in this study employed a random sampling method in which students from university housing were selected. The studies involving human participants were reviewed and approved by The Research Ethics Committee of Southwestern University of Finance and Economics. The participants provided their written informed consent to participate in this study. The sample procedures follow. Each student apartment complex was numbered, including five apartment buildings for post-graduates and 11 buildings for undergraduates. Each dormitory in each complex was numbered and 350 random numbers generated by creating random samples in Microsoft Excel. All of the students in a selected dormitory were surveyed; in total, 1,065 questionnaires were distributed and 1,027 retrieved, from which 872 were valid, a recorded validity of 87.43%.

Stata 17.0 software was used to record the participants’ basic information and mean test of social efficacy in different groups, and then the Dummy Variable Regression Model was adopted, treating the general, interpersonal, and academic self-efficacy scores as the dependent variables (continuous variables), the different groups as the explanatory variables, and the personal and family factors as control variables by which the differences in self-efficacy in different social activity groups were investigated. Eviews 7.2 was used for the regression models.

## Results

### Descriptive Statistics

#### Basic Information About the Survey Participants

Table 1 shows 30.39% of the subjects (265 students) were male and 69.61% of the subjects (607 students) female. The ratio of males to females in the sampled university is approximately 3:7, which is true of the surveyed universities. Among the sample participants, 439 students considered themselves to be extroverts and 433 introverts. The number of sampled students who spent no fewer than 3 h online was 500, far surpassing the number of those that spent fewer than 3 h online by 372. Fathers of 442 of the sampled students have secondary education certificates, 357 hold college and higher degrees, and 73 received no more than elementary education. The number of university students with household monthly incomes below ¥3,000, in the range ¥3,000–¥7,000, in the range ¥7,000–¥10,000, and above ¥10,000, was 101, 348, 228, and 185, respectively.

**TABLE 1:** Basic participant information.

Personal information	Frequency (%)	Places of household registration	Frequency (%)	Paternal education	Frequency (%)
Male	265 (30.39)	Eastern regions	232 (26.61)	Primary School or Below	73 (8.37)
Female	607 (69.61)	Central regions	171 (19.61)	Secondary level	442 (50.69)
Extrovert	439 (50.34)	Western regions	469 (53.78)	College or above	357 (40.94)
Introvert	433 (49.65)	**Areas**	**Frequency (%)**	Household monthly family income	**Frequency (%)**
Time spent online no less than 3 h	500 (57.34)	Urban areas	258 (29.58)	Below ¥3,000	101 (11.71)
Time spent online 3 h or below	372 (42.66)	Rural areas	614 (70.42)	¥3,000-¥7,000	348 (40.37)
				¥7,000-¥10,000	228 (26.45)
				Above ¥10,000	185 (21.46)

As the data show, 258 students were from urban areas, accounting for 29.58% of the sampled students, whereas 614 were from rural areas, accounting for 70.42% of the total. Approximately 26.61% of the sampled students were from eastern China (232), 19.61% from central China (171), and 53.78% from western China (469). The ratio agrees with the circumstances of the surveyed university, which is located in western China.

#### The Social Activity of University Students

The social activity of university students were classified based on their self-reported scores of social life in virtual and physical spaces. According to the online and offline social activity frequency distribution, approximately 60% of the overall scores were utilized as the benchmark to determine one’s level of activity, either in the real or virtual worlds. A score of 42 or above was considered socially active. A score below 42 indicated the participant was socially inactive. “Rich-get-richer” denotes the group of students socially active in the virtual space (with an online social score greater than 42) and in the physical space (with an offline social score greater than 42). “Online social weakness” refers to students not active in the virtual space (with an online social score below 42) but active in the physical space (with an offline social score greater than 42). “Poor-get-poorer” describes those neither active online nor offline (with a score below 42 for both virtual and physical spaces). Students who are active online (with an online score greater than 42) but inactive offline (with a score below 42 in the physical sphere) belong to the “social compensation” group.

Table 2 shows the classifications of online and offline social activity of the sampled university students. Judging from the social activity, an overwhelming majority of students were found to be active online. The number of students with an online score greater than 42 was 695, accounting for 80% of survey participants, while 55.44% of students were active in offline settings. According to these results, it appears that online social activity has overtaken offline social networking as the mainstream mode of social interaction at the surveyed universities.

**TABLE 2:** Types of social activity.

Types percentage	Rich-get-richer	Online social weakness	Poor-get-poorer	Social compensation
Criterion	Offline social score ≥ 42 Offline social score ≥ 42	Online social score < 42 Offline social score ≥ 42	Online social score < 42 Offline social score < 42	Online social score ≥ 42 Offline social score < 42
*n*	436	47	130	259
Percentage	50.05%	5.39%	14.93%	29.73%

Judging by the social activity, more than one-third of university students have inconsistent levels of online and offline social behavior. The data collected in this study suggest that 436 students fall into the “rich-get-richer” group, 259 into the “social compensation” group, 130 into the “poor-get-poorer” group, and 130 into the “online social weakness” group. The “social compensation” group outweighs the population of groups exhibiting inconsistent online and offline social performance, and “online social weakness” only forms a very small proportion of the total.

### Comparative Analyses of Inconsistencies in Social Behavior and Self-Efficacy of University Students

#### Mean Test of Self-Efficacy of University Students With Different Levels of Social Activity

The self-efficacy of university students was assessed based on their scores on the provided scales. The upper portion of Table 3 lists the mean of the university students’ general and task-specific self-efficacy scores. Compared across the groups, the self-efficacy of students varies with social activity and the variation is consistent within groups. The self-efficacy of the “social compensation” group is higher than that of “online social weakness.” It can also be seen that the highest self-efficacy is reported for the “rich-get-richer” group, which confirms the existing research hypotheses.

**TABLE 3:** Self-efficacy mean value of students with different social activity and testing results.

Types of social activity	General self-efficacy	Social self-efficacy		Academic self-efficacy
Rich-get-richer	47.64	37.59		38.00
Social compensation	44.05	35.14		36.76
Online social weakness	42.94	32.23		35.26
Poor-get-poorer	38.50	29.54		33.16

**Scales of self-efficacy**	**Variable**	**Mean difference**		**SD**
	**(I) Social activity type (J)**	**Social activity type (I) – (J)**		

General self-efficacy	Rich-get-richer	Social compensation	3.60***	0.612
		Online social weakness	4.70***	1.21
		Poor-get-poorer	9.14***	0.79
Social self-efficacy	Rich-get-richer	Social compensation	2.45***	0.71
		Online social weakness	5.36***	1.39
		Poor-get-poorer	8.05***	0.90
Academic self-efficacy	Rich-get-richer	Social compensation	1.24***	0.60
		Online social weakness	2.75***	1.17
		Poor get poorer	4.84***	0.76

***P < 0.01; **P < 0.05; *P < 0.1.

Compared with social self-efficacy, academic self-efficacy of all the groups was higher, with a score above 33. A significant difference emerged between the social efficacy of the different groups though small differences in self-efficacy between the groups. The lower portion of Table 3 presents the results of the mean test of self-efficacy. With the “rich-get-richer” group as the reference group, the score difference of self-efficacy across the groups was verified, confirming significant differences in self-efficacy across the groups. On one hand, small differences exist in academic self-efficacy between the groups, on the other hand, a significant difference emerged between the social efficacy of the different groups, with the “poor-get-poorer” group scoring 8 points lower than the “rich-get-richer” group.

#### Relationship Between Social Activity Types and Self-Efficacy

As the results show, the explanatory power of all of the models falls somewhere in the interval 11–17%, indicating moderately convincing results. Naturally, there are vast differences between the general and specific-task self-efficacy between the different groups: social activity has the dominant explanatory power for self-efficacy differences, compared with regional, individual, and family factors.

With university students’ general self-efficacy as the analysis variable, the regression results of model 1, listed in Table 4, show that significant differences emerge for general self-efficacy with different levels of social activity, the overriding factor accounting for the differences. The “rich-get-richer” group scored the highest and the “poor-get-poorer” group the lowest, with the values of “rich-get-richer,” “social compensation,” and “online social weakness” surpassing “poor-get-poorer” by score factors of 8.95, 5.57, and 4.41, respectively. Of the two groups showing inconsistent social behavior, the “social compensation” group had higher general self-efficacy than the “online networking weakness” group, but it lagged far behind the “rich-get-richer” group. Hypothesis 1 was supported.

**TABLE 4:** Types of university students’ social activities and model of differences in self-efficacy.

		General self-efficacy	Academic self-efficacy	Social self-efficacy
				
		Model 1	Model 2	Model 3
Social activity type Poor-get-poorer (Reference group)	Rich-get-richer	8.95*** (11.25)	7.67*** (8.42)	5.07*** (6.52)
	Social compensation	5.57** (6.56)	5.47*** (5.62)	3.86*** (4.64)
	Online social weakness	4.41*** (3.33)	2.58* (1.70)	2.07 (1.60)
Regional factor	Central China	0.27 (0.47)	0.22 (0.34)	−0.02 (−0.04)
Western China (Reference group)	Eastern China	1.22* (1.92)	0.76 (1.03)	1.13* (1.80)
Individual factor	Male (reference group: female)	1.12** (2.08)	1.67** (2.54)	0.73 (1.31)
	Extraverts (reference group: introverts)	1.86*** (3.67)	2.62*** (4.48)	0.28 (0.56)
	Time spent online per day more than 3 h (reference group: Time spent online per day below 3 h)	0.28 (0.53)	0.16 (0.27)	1.01* (1.93)
Family factor	Paternal education (reference group: no more than elementary level)			
	Secondary level	−0.33 (−0.31)	−0.04 (−0.03)	−0.23 (−0.22)
	College and above	1.34 (1.11)	1.56 (1.12)	1.70 (1.43)
	Household monthly income (reference group: below ¥3,000)			
	¥3,000–¥7,000	−1.53** (−2.97)	−0.91** (−1.54)	−1.34** (2.66)
	¥7,000–¥10,000	0.22 (0.41)	0.03 (0.04)	0.27 (0.50)
	Above ¥10,000	−0.11 (−0.18)	−0.21 (−0.31)	−0.55 (−0.94)
Adjusted *R*^2^		16.46%	11.53%	15.71%
*F*-statistic		11.07	7.66	9.10
D—W stat		1.92	1.98	1.84

***P < 0.01; **P < 0.05; *P < 0.1; within the parentheses is the value of *t.*

The data in model 2 show the academic self-efficacy of university students with different levels of social activity to be significantly different: the score for the “social compensation” group is higher than that of the “online social weakness” group. To be precise, the academic self-efficacies of the “rich-get-richer,” “social compensation,” and “online social weakness” groups are, respectively, 7.67, 5.47, and 2.58 points higher than those of the “poor-get-poorer” group. The data from model 3 suggest that there is no significant difference in social self-efficacy between the “online social weakness” and “poor-get-poorer” groups, both of which are low-scoring groups, and that the scores for the “social compensation” and “rich-get-richer” groups are larger than those for the “poor-get-poorer” group by 3.86 and 5.07 points, respectively.

The data from models 2 and 3 show that the ratings for academic self-efficacy are significantly different between the four groups of students, although the difference in social self-efficacy is less significant, and the two groups who are less active online scored the least in social self-efficacy. The ranking of the social self-efficacy of the groups is “rich-get-richer” > “social compensation” > “online network weakness” = “poor-get-poorer,” Hypothesis 2 was partially supported.

## Conclusion and Discussion

Many researchers have analyzed how offline social behavior influences online social behavior; however, conflicts in social behavior between physical and virtual spaces have drawn little attention. To help explain this phenomenon, in this study, a university student social typology comprising four types of social activity was constructed by utilizing characteristics of social behavior in both online and offline spheres. On the basis of social activity types, the differences in self-efficacy across the groups studied were compared, especially those with inconsistencies in their online and offline social behavior.

First, the results suggest that the widespread use of social media has led to the prominent emergence of different types of social activity, and inconsistent online and offline social behavior has become commonplace. More than half of university students surveyed rated themselves as being socially active both online and offline, and barely 15% indicated that they are generally socially inactive in both environmental settings. Over 80% are socially active online, which is approximately twice the ratio of those who are active offline. The majority of the one-third who showed inconsistent online and offline social behavior fall into the “social compensation” group, the online activity of which eclipses their social experiences offline; only a few students are active online but inactive offline. These results illustrate a picture of the university environment in which online social networking has become the dominant form of social interaction.

Second, the self-efficacy of students with inconsistent social behavior is significantly different from that of those with consistent social behavior; among students with inconsistent social behavior, the self-efficacy of students socially active online is higher than those socially active offline. Of the four types, students with consistent behavior are either at the high end of the scale (active online and offline) or at the low end of the scale (inactive online and offline). Self-efficacy was evaluated from the perspective of general and task-specific (academic and social) self-efficacy; the results show that social activity plays a dominant role in self-efficacy compared with other factors. Based on analysis of the data collected, it can also be verified that previously held assumptions about social behavior, e.g., personality, sex, and socioeconomic status, exert some effects on university students’ self-efficacy (Amichai-Hamburger and Vinitzky, 2010), although social activity plays the dominant role. Social inactivity online was the most prominent predictor of low social self-efficacy. The result also show unexpected results that there are no significant difference between the “online network weakness” group and “poor-get-poorer” group on social efficacy. It may be mirrored inactive online social has stronger tie with low social efficacy than active online social.

Third, regarding task-specific self-efficacy (the academic and social varieties), it was found that the gaps between different social group types were larger than those in the self-assessment of social self-efficacy. Although the mean test suggests that the differences for the academic self-efficacy of different groups is smaller than those for social self-efficacy, the results of the model described herein show that, after controlling related variables, the difference in the scores related to academic self-efficacy between the different social activity groups was more significant than those for social self-efficacy. Though social activity groups were categorized based on generalized types of social interaction, for academically stressed students, the score difference in academic self-efficacy of different social activity groups was found to be larger when compared with social self-efficacy, a finding that indicates a stronger association of university students’ social relations with academic performance. Amicable social interactions can enhance students’ academic achievements, a conclusion that resonates with existing research (Meng et al., 2012).

Meanwhile, the negative effects of online networking, such as mood swings, decreased attention span, and insensitivity to or ignorance of their family’s feelings (Wen et al., 2016; Aniko et al., 2017), cannot go unnoticed or unreported. Existing research has identified negative behaviors associated with online social behavior, e.g., becoming more prone to depressive moods with longer screen time. Additionally, others’ online self-presentations are likely to foster upward social comparison and inaccurate judgment about oneself (Bessière et al., 2008; Boers et al., 2019), a phenomenon that has negative real-life consequences due to excessive use of social media.

In the present study, university students’ online-offline social behavior and self-efficacy was probed. From the findings, it seems useful that university students capitalize on online social interactions, a novel carrier of self-efficacy, not only to participate in modern social life, but especially to surmount social barriers such as social anxiety disorder or stereotyping, and to compensate for resulting inadequacies in their offline social interactions so as to enhance their self-efficacy and boost their ability to learn and live.

## Limitation and Future Directions

Several limitations of this study are worth mentioning. First, the self-reported questionnaire of media use may not conform to actuality, e.g., that light users tend to over-report while heavy users tend to under-report (Deng et al., 2019), which involves measurement errors (Araujo et al., 2017). However, new research has revealed that the effect sizes of correlations using self-reported data tend to be smaller compared to those using logged data (Jones-Jang et al., 2020).

Second, extroversion/introversion should be tester by scale, and extroversion/introversion patterns of behavior should be find out before this observation. For extroversion contribute to sharing more emotion (Pentina and Zhang, 2017), study on this should be more deeper thought should be devoted to the naming of “poor-get-poorer” “online social weakness” social activity types. Such as the “poor-get-poorer” group is named as an opposite of that based on “rich-get-richer” theory, but not every student want to get “richer.” The names may not fully reflect the reality of the groups and should be adjusted in follow-up studies.

Third, the purpose to use social media and social media platforms are not distinguished from each other. Social media usage for educational purposes positively related to academic performance (Boahene et al., 2019), some college student use social media to get message and someone just use it to sociality and get recognition. This different may has separate relationship with self-efficacy. Some require that users have a “friend” relationship to see posts and to communicate, e.g., WeChat and QQ, while others do not have such requirements, e.g., Weibo, the Chinese version of Twitter. Online social interactions may involve offline friends and strangers (Blais et al., 2007; Kahai and Lei, 2019), but the interactions may be different. And different platform may operate differently and social media usage may positive direct effect or negative direct effect to the self-efficacy (Jenna et al., 2020).

Several points will be addressed in future studies. Social activity was classified based on observation, but in-depth empirical analyses were not conducted and the root cause of behavioral inconsistencies was not verified. From this evaluation, group characteristics were summarized, but the question of why students with inconsistent online and offline social behavior tend to use a specific way of interacting remains, as does that of whether such students have anything in common. Future work includes determining why social behavior is apparently more closely related to academic self-efficacy than to social self-efficacy. Therefore, a follow-up study is planned in which the motivations and root causes behind said inconsistencies will be examined and the analyses of the relationship between university students’ social interactions and self-efficacy thereby enriched.

## Data Availability Statement

The raw data supporting the conclusions of this article will be made available by the authors, without undue reservation.

## Ethics Statement

The studies involving human participants were reviewed and approved by the Research Ethics Committee of Southwestern University of Finance and Economics. The participants provided their written informed consent to participate in this study.

## Author Contributions

YY, YD, and HY conceived of the presented idea. YY and YD designed the methods of data collection, performed the data analysis, and wrote the first draft of the manuscript. YY and HY commented on and revised the drafts. All authors contributed to the article and approved the submitted version.

## Conflict of Interest

The authors declare that the research was conducted in the absence of any commercial or financial relationships that could be construed as a potential conflict of interest.
