# Supplementary material for: Comparative Study on Relationship Between Inconsistent Online-Offline Social Performance and Self-Efficacy of University Students Based on Types of Social Activity
Source: Front Psychol. 2021 Mar 15;12:603971. doi: 10.3389/fpsyg.2021.603971 (PMC8005636; doi:10.3389/fpsyg.2021.603971)
Supplement: Supplementary file 1 [file Data_Sheet_1.docx]

**1：社交活跃度量表**

**1.Social Activity Scales**

**“1——7”代表了你对该问题的同意程度，1为非常不同意，7为非常同意 请根据实际情况对该问题做出回答，在相印的数字上打√即可**

Below is a list of statements. To what extent do you agree or disagree with these statements? Your answer could be a score between 1 and 7 (1 for strongly disagree,7 for strongly agree).Below is a list of statements. To what extent do you agree or disagree with these statements? Your answer could be a score between 1 and 7 (1 for strongly agree,7 for strongly disagree). Below is a list of statements. To what extent do you agree or disagree with these statements? Your answer could be a score between 1 and 7 (1 for strongly agree,7 for strongly disagree). Below is a list of statements. To what extent do you agree or disagree with these statements? Your answer could be a score between 1 and 7 (1 for strongly agree,7 for strongly disagree).

| **现实生活中的情况( offline life )** |  |  |  |  |  |  |  |
| --- | --- | --- | --- | --- | --- | --- | --- |
| C1.我会和朋友分享生活见闻  （I often share my life experiences with my friends） | 1 | 2 | 3 | 4 | 5 | 6 | 7 |
| C2.我很注意自己的形象  （I care about my image） | 1 | 2 | 3 | 4 | 5 | 6 | 7 |
| C3.我有很多朋友  （I have lots of friends） | 1 | 2 | 3 | 4 | 5 | 6 | 7 |
| C4.我经常参加社会活动（包括社会实践、社团活动等）  I often take part in social activities (including social practice, club activities, etc.) | 1 | 2 | 3 | 4 | 5 | 6 | 7 |
| C5.我经常和朋友分享我的心情  （I often share my feelings with my friends） | 1 | 2 | 3 | 4 | 5 | 6 | 7 |
| C6.朋友经常和我分享他们的心情  （My friends often share their feelings with me） | 1 | 2 | 3 | 4 | 5 | 6 | 7 |
| C7.我经常和朋友聊一些社会问题。  I often discuss social issues with my friends. | 1 | 2 | 3 | 4 | 5 | 6 | 7 |
| C8.我在很多活动中担任重要职位  I play important role in many social activities | 1 | 2 | 3 | 4 | 5 | 6 | 7 |
| C9.我和同学的关系比较融洽  I get on well with my classmates | 1 | 2 | 3 | 4 | 5 | 6 | 7 |
| C10.我经常和朋友聚会、聚餐  I often party or dine with my friends |  |  |  |  |  |  |  |

| **社交网络中的情况( social meadia)** |  |  |  |  |  |  |  |
| --- | --- | --- | --- | --- | --- | --- | --- |
| D1.我会在社交网络上分享自己的心情  I share my feelings on social media | 1 | 2 | 3 | 4 | 5 | 6 | 7 |
| D2.我在网上发的照片都是精心挑选的  The photos I post online are carefully selected | 1 | 2 | 3 | 4 | 5 | 6 | 7 |
| D3.我在社交网络上有很多好友  I have a lot of friends on social media | 1 | 2 | 3 | 4 | 5 | 6 | 7 |
| D4.我经常通过社交网络关注社会动态与热点新闻  I often follow social trends and hot news through social media | 1 | 2 | 3 | 4 | 5 | 6 | 7 |
| D5.我经常分享对某些社会焦点的看法  I often share my views on certain social concerns on social media | 1 | 2 | 3 | 4 | 5 | 6 | 7 |
| D6.我经常对好友发布的内容点赞和评论  I often comment on or thumb up my friends posts. | 1 | 2 | 3 | 4 | 5 | 6 | 7 |
| D7.朋友会经常对我发布的内容点赞和评论  My friends often comment on or thumb up my posts | 1 | 2 | 3 | 4 | 5 | 6 | 7 |
| D8.我会常常更新自己在社交网络的状态  I often update my status on social lmedia | 1 | 2 | 3 | 4 | 5 | 6 | 7 |
| D9.我喜欢在社交网络上和别人沟通交流  I like to communicate with others on social media | 1 | 2 | 3 | 4 | 5 | 6 | 7 |
| D10.我经常在朋友群里发言  I am active in group chat. |  |  |  |  |  |  |  |

**2：自我效能感量表**

**2.Social Efficacy Scales**

**“1——5”代表了你对该问题的同意程度，1为非常不同意，5为非常同意 请根据实际情况对该问题做出回答，在相印的数字上打√即可**

**Below is a list of statements. To what extent do you agree or disagree with these statements? Your answer could be a score between 1 and 5 (1 for strongly agree, 5for strongly disagree).**

| **人际交往方面( interpersonal communication)** |  |  |  |  |  |
| --- | --- | --- | --- | --- | --- |
| E1.只要我愿意，我总能交到朋友  I can always make friends if I hope to | 1 | 2 | 3 | 4 | 5 |
| E2.即使对方与我有矛盾，我也能找到解决的办法  I can always find a solution to get on well with a person even if he/she has trouble with me, | 1 | 2 | 3 | 4 | 5 |
| E3.对我而言，处理人际关系很轻松  For me, dealing with interpersonal relationships are easy | 1 | 2 | 3 | 4 | 5 |
| E4.我能够与不同类型的人打好交道  I can get on well with different types of people | 1 | 2 | 3 | 4 | 5 |
| E5.我能够应付突如其来的人际矛盾和纠纷  I can cope with the unexpected interpersonal conflicts and disputes | 1 | 2 | 3 | 4 | 5 |
| E6.即使身处复杂的交往圈中，我也相信自己有能力应对  I am confident to cope with a complicated social circle | 1 | 2 | 3 | 4 | 5 |
| E7.当和别人发生矛盾时，我通常会想到解决的  I usually come up with a solution when I having a problem with someone | 1 | 2 | 3 | 4 | 5 |
| E8.即使有突发事件，我也能很好地处理人际关系  I can handle the interpersonal relationship well under an unforeseen circumstances | 1 | 2 | 3 | 4 | 5 |

| **学习方面(academic)** | 非常不同意 | 不同意 | 中立 | 同意 | 非常同意 |
| --- | --- | --- | --- | --- | --- |
| E9.只要认真学习，我就能取得好成绩  As long as I study hard, I can get good grades | 1 | 2 | 3 | 4 | 5 |
| E10.即使当时学不懂，我也能想办法弄明白  Even if I had difficulties in the learning process, I can always figure it out later | 1 | 2 | 3 | 4 | 5 |
| E11.我都能达到预设的学习目标  I can always achieve my preset academic goals | 1 | 2 | 3 | 4 | 5 |
| E12.我相信我能应付任何学习困难  I'm confident to cope with any academic difficulties | 1 | 2 | 3 | 4 | 5 |
| E13.以我的成绩，可应付各类临时考试  With my academic abilityl, I am able to deal with all kinds of temporary examinations | 1 | 2 | 3 | 4 | 5 |
| E14.当学习烦躁时，我会找到自我调节的办法  I can find a way to adjust myself when I feel fretful during study | 1 | 2 | 3 | 4 | 5 |
| E15.当学习遇到困难时，我会想到办法解决  I can always find a solution to solve difficulties in study | 1 | 2 | 3 | 4 | 5 |
| E16.无论难度有多大，我也能学好要求的知识  I can always learn well as required no matter how difficult it is | 1 | 2 | 3 | 4 | 5 |
