# Supplementary material for: Comparative Study on Relationship Between Inconsistent Online-Offline Social Performance and Self-Efficacy of University Students Based on Types of Social Activity
Source: Front Psychol. 2021 Mar 15;12:603971. doi: 10.3389/fpsyg.2021.603971 (PMC8005636; doi:10.3389/fpsyg.2021.603971)
Supplement: Supplementary file 2 [file Data_Sheet_2.docx]

The factor analysis results of social activity

1.offline social activity: relationship building（C2 C3 C4 C8 C9）+ relationship retention（C1 C5 C6 C7 C10）

1) offline relationship building（C2 C3 C4 C8 C9）.

the value of Kaiser-Meyer-Olkin is .740. and get one component.

| **Table 1-1 KMO and Bartlett's Test** | | |
| --- | --- | --- |
| Kaiser-Meyer-Olkin Measure of Sampling Adequacy. | | .740 |
| Bartlett's Test of Sphericity | Approx. Chi-Square | 826.140 |
|  | df | 10 |
|  | Sig. | .000 |

**Table 1-2 Total Variance Explained**

| Component | Initial Eigenvalues | | | Extraction Sums of Squared Loadings | | |
| --- | --- | --- | --- | --- | --- | --- |
|  | Total | % of Variance | Cumulative % | Total | % of Variance | Cumulative % |
| 1 | 2.313 | 46.256 | 46.256 | 2.313 | 46.256 | 46.256 |
| 2 | 1.045 | 18.903 | 65.158 |  |  |  |
| 3 | .647 | 12.949 | 80.107 |  |  |  |
| 4 | .541 | 10.810 | 90.917 |  |  |  |
| 5 | .454 | 9.083 | 100.000 |  |  |  |

| **Table 1-3 Component Matrixa** | |
| --- | --- |
|  | Component |
|  | 1 |
| C9 | .780 |
| C3 | .774 |
| C2 | .772 |
| C8 | .691 |
| C4 | .580 |
| Extraction Method: Principal Component Analysis. | |
| a. 1 components extracted. | |

2) offline relationship retention（C1 C5 C6 C7 C10）. the value of Kaiser-Meyer-Olkin is .684. and get one component.

| **Table 2-1 KMO and Bartlett's Test** | | |
| --- | --- | --- |
| Kaiser-Meyer-Olkin Measure of Sampling Adequacy. | | .684 |
| Bartlett's Test of Sphericity | Approx. Chi-Square | 653.580 |
|  | df | 10 |
|  | Sig. | .000 |

**Table 2-2 Total Variance Explained**

| Component | Initial Eigenvalues | | | Extraction Sums of Squared Loadings | | |
| --- | --- | --- | --- | --- | --- | --- |
|  | Total | % of Variance | Cumulative % | Total | % of Variance | Cumulative % |
| 1 | 2.109 | 42.177 | 42.177 | 2.109 | 42.177 | 42.177 |
| 2 | 1.083 | 19.656 | 61.833 |  |  |  |
| 3 | .759 | 15.186 | 79.019 |  |  |  |
| 4 | .590 | 11.805 | 90.824 |  |  |  |
| 5 | .459 | 9.176 | 100.000 |  |  |  |

| **Table 2-3 Component Matrix^a^** | |
| --- | --- |
|  | Component |
|  | 1 |
| C1 | .785 |
| C6 | .695 |
| C10 | .674 |
| C5 | .603 |
| C7 | .583 |
| Extraction Method: Principal Component Analysis. | |
| a. 1 components extracted. | |

2.Online social activity: communication（D9 D10 D6 D7）+broadcasting（D1 D2 D3 D4 D5 D8）

1)Online social activity：communication（D9 D10 D6 D7）

the value of Kaiser-Meyer-Olkin is 0.601,and get one component.

| **Table 3-1 KMO and Bartlett's Test** | | |
| --- | --- | --- |
| Kaiser-Meyer-Olkin Measure of Sampling Adequacy. | | .601 |
| Bartlett's Test of Sphericity | Approx. Chi-Square | 321.543 |
|  | df | 6 |
|  | Sig. | .000 |

| **Table 3-2 Total Variance Explained** | | | | | | |
| --- | --- | --- | --- | --- | --- | --- |
| Component | Initial Eigenvalues | | | Extraction Sums of Squared Loadings | | |
|  | Total | % of Variance | Cumulative % | Total | % of Variance | Cumulative % |
| 1 | 1.743 | 43.578 | 43.578 | 1.743 | 43.578 | 43.578 |
| 2 | .971 | 24.268 | 67.845 |  |  |  |
| 3 | .720 | 17.999 | 85.844 |  |  |  |
| 4 | .566 | 14.156 | 100.000 |  |  |  |

| **Table 3-3 Component Matrix^a^** | |
| --- | --- |
|  | Component |
|  | 1 |
| D6 | .733 |
| D10 | .699 |
| D9 | .629 |
| D7 | .567 |
| Extraction Method: Principal Component Analysis. | |
| a. 1 components extracted. | |

2)Online social activity：broadcasting（D1 D2 D3 D4 D5 D8）

the value of Kaiser-Meyer-Olkin is 0.702, and get one component.

| **Table 4-1 KMO and Bartlett's Test** | | |
| --- | --- | --- |
| Kaiser-Meyer-Olkin Measure of Sampling Adequacy. | | .728 |
| Bartlett's Test of Sphericity | Approx. Chi-Square | 528.839 |
|  | df | 10 |
|  | Sig. | .000 |

| **Table 4-2 Total Variance Explained** | | | | | | |
| --- | --- | --- | --- | --- | --- | --- |
| Component | Initial Eigenvalues | | | Extraction Sums of Squared Loadings | | |
|  | Total | % of Variance | Cumulative % | Total | % of Variance | Cumulative % |
| 1 | 2.086 | 41.719 | 41.719 | 2.086 | 41.719 | 41.719 |
| 2 | .894 | 17.889 | 59.608 |  |  |  |
| 3 | .790 | 15.808 | 75.416 |  |  |  |
| 4 | .712 | 14.231 | 89.647 |  |  |  |
| 5 | .632 | 12.378 | 94.674 |  |  |  |
| 6 | .518 | 10.353 | 100.000 |  |  |  |

| **Table 4-3 Component Matrix^a^** | |
| --- | --- |
|  | Component |
|  | 1 |
| D1 | .785 |
| D3 | .695 |
| D2 | .674 |
| D4 | .603 |
| D8 | .583 |
| D5 | .439 |
| Extraction Method: Principal Component Analysis. | |
| a. 1 components extracted. | |
